# Characterization and Benzo[a]pyrene Content Analysis of *Camellia* Seed Oil Extracted by a Novel Subcritical Fluid Extraction

**DOI:** 10.1007/s11746-013-2293-1

**Published:** 2013-07-10

**Authors:** Jianyin Miao, Ke Che, Ruchun Xi, Liping He, Xuexiang Chen, Xiaosheng Guan, Xueying Zhuang, Xiujun Wen, Yong Cao

**Affiliations:** 1 College of Food Science, South China Agricultural University, No. 483 Wushan Road, Guangzhou, 510642 People’s Republic of China; 2 College of Forestry, South China Agricultural University, No. 483 Wushan Road, Guangzhou, 510642 People’s Republic of China; 3 College of Ocean, Qinzhou University, Qinzhou, 535000 People’s Republic of China

**Keywords:** Subcritical *n*-butane extraction, *Camellia* seed oil, Fatty acid composition, Physicochemical properties, Benzo[a]pyrene

## Abstract

A novel continuous subcritical *n*-butane extraction technique for *Camellia* seed oil was explored. The fatty acid composition, physicochemical properties, and benzo[a]pyrene content of *Camellia* seed oil extracted using this subcritical technique were analyzed. Orthogonal experiment design (L_9_(3^4^)) was adopted to optimize extraction conditions. At a temperature of 45 °C, a pressure of 0.5 MPa, a time of 50 min and a bulk density of 0.7 kg/L, an extraction yield of 99.12 ± 0.20 % was obtained. The major components of *Camellia* seed oil are oleic acid (73.12 ± 0.40 %), palmitic acid (10.38 ± 0.05 %), and linoleic acid (9.15 ± 0.03 %). Unsaturated fatty acids represent 83.78 ± 0.03 % of the total fatty acids present. Eight physicochemical indexes were assayed, namely, iodine value (83.00 ± 0.21 g I/100 g), saponification value (154.81 ± 2.00 mg KOH/g), freezing-point (−8.00 ± 0.10 °C), unsaponifiable matter (5.00 ± 0.40 g/kg), smoke point (215.00 ± 1.00 °C), acid value (1.24 ± 0.03 mg KOH/g), refrigeration test (transparent, at 0 °C for 5.5 h), and refractive index (1.46 ± 0.06, at 25 °C). Benzo[a]pyrene was not detected in *Camellia* seed oil extracted by continuous subcritical *n*-butane extraction. In comparison, the benzo[a]pyrene levels of crude *Camellia* seed oil extracted by hot press extraction and refined *Camellia* seed oil were measured at 26.55 ± 0.70 and 5.69 ± 0.04 μg/kg respectively.

## Introduction

*Camellia*, which belongs to *Camellia oleifera* plants rich in oil, have been cultivated in China for more than 2000 years. *C. oleifera* plants are mainly distributed in south China (3.5 million hm^2^), and produce about 5.6 million tons of *Camellia* seeds each year [1]. *Camellia* seed oil, which is also called oriental olive oil, is recommended as a health-care plant oil by FAO because of its high content of unsaturated fatty acids, polyphenols, vitamin E and carotene.

Traditional processing methods such as hot press extraction and organic solvent extraction are generally applied to obtain the seed oil [2]. Supercritical fluid extraction is also attracting increasing attention because of its advantages of high yield and superior oil quality [3–5]. There are negative aspects to all three processing methods: traditional hot press extraction results in lower than desired yields, and organic solvent extraction is associated with environmental concerns; supercritical fluid extraction requires high pressures and is expensive, limiting its industrial application.

Recently, the presence of benzo[a]pyrene, a known carcinogen, in some commercially available *Camellia* seed oils has aroused widespread attention in society (http://finance.ifeng.com/news/special/jhcy/20100903/2586740.shtml), and the effect of different production processes on benzo[a]pyrene content in *Camellia* seed oil has become a focus of research. High temperature processing during both hot press and solvent extraction production techniques is considered to be a main cause of excessive benzo[a]pyrene formation [6]. Therefore, a more efficient, safer, and low-cost technique for extracting *Camellia* seed oil is needed.

Subcritical fluid extraction is performed at lower temperatures and pressures than those employed in supercritical fluid extraction. Subcritical fluid extraction is a continuous counter current process, After extraction, the solvent is removed using a vacuum at a low temperature. The subcritical fluid extraction process is safe and efficient, and does not damage the heat-sensitive components of the materials nor does it result in formation of benzo[a]pyrene. As there are few reports on the novel extraction of *Camellia* seed oil from *Camellia* seeds, the purpose of this study was to determine optimal continuous subcritical fluid extraction conditions for the production of *Camellia* seed oil. The study employed orthogonal experimental design and the parameters investigated in the extracted oil were fatty acid composition by gas chromatography–mass spectrometry (GC–MS), physicochemical properties, and benzo[a]pyrene content by high-performance liquid chromatography (HPLC).

## Materials and Methods

### Materials and Reagents

*Camellia* seeds, which were cultivated in Hunan Province, were supplied by Jing Hui Forestry Technology Development Center (Hengyang, China). After picking, drying and dehulling, the seed samples were stored at 4 °C in polythene bags until extraction. Crude *Camellia* seed oil and refined *Camellia* seed oil extracted by hot press extraction were supplied by Guangdong Taiyuan Nongke Co., Ltd. (Meizhou, China).

*n*-Butane solvent (99.99 %) was purchased from Shenzheng Shenyan Gas Co., Ltd. (Shenzhen, China). Benzo[a]pyrene was supplied by the Guangzhou Bureau of Quality and Technical Supervision (Guangzhou, China). Other chemicals were of analytical grade.

### Preparation of *Camellia* Seed Oil by Continuous Subcritical *n*-Butane Fluid Extraction

The continuous subcritical fluid extractor used in the experiments was constructed by our laboratory. The extractions were conducted in a 5-L extraction vessel. Before extraction, the *Camellia* seeds were ground into a powder (10 mesh) and dried at 55 °C for 4–6 h to ensure moisture levels of <4 %. Based on single factor experiments, an orthogonal design L_9_(3^4^) was conducted to optimize the extraction conditions (Table 1). The extraction yield was calculated with the following formula:

$$ {\hbox{Extraction yields}} = \frac{{\hbox{The total oil in raw material}} - {\hbox{the oil in extracted residue}}}{\text{The total oil in raw material}} \times 100\,\%. $$

**Table 1:** Primary variables and levels used in the orthogonal design

Levels	Main variables			
	Pressure (A, MPa)	Time (B, h)	Temperature (C, °C)	Bulk density (D, kg/L)
1	0.4	30	35	0.6
2	0.5	50	40	0.7
3	0.6	70	45	0.8

### Analysis of Fatty Acids by GC–MS

Before GC–MS analysis, the fatty acids of the experimental *Camellia* seed oil were derivatized into methyl esters [4, 7]. 50 mg of *Camellia* seed oil was weighed into a stoppered test tube and dissolved in 2 mL petroleum ether. Two (2) mL of 0.4 mol/L potassium hydroxide methanol solution was then added to the test tube, stirred, and held at room temperature for 30 min. Finally, distilled water was added and the organic phase was injected into a GC–MS system (Agilent Co., USA) for analysis. Qualitative analysis was performed using the standard peak retention times of fatty acids and the mass spectra library. Quantitative analysis of fatty acids was determined by measuring peak area.

The GC–MS conditions were set as follows: column oven initial temperature was 100 °C, programmed from 100 to 230 °C at 10 °C/min, and held for 40 min. The temperatures of the quadrupoles, the ion source chamber, and the split injector were held at 150, 230, and 250 °C, respectively. Helium was used as the carrier gas, and had a split ratio of 10:1.

### Determination of the Physicochemical Properties

The physicochemical properties of the experimental *Camellia* seed oil were also measured, These included acid value, iodine value, saponification value, unsaponifiable matter, refractive index, freezing point, refrigeration test and smoke point, and were assayed according to the Chinese Pharmacopeia (2005) and GB 11765-2003 (a Chinese detection techniques and quality standard for the physicochemical properties detection of *Camellia* seed oil*)*. All assays were carried out in triplicate.

### Analysis of Benzo[a]pyrene Content by HPLC

The analysis of benzo[a]pyrene in *Camellia* seed oil was carried out according to the guidelines published by Wu et al. [6] and GB/T 5009.27-2003 (a Chinese detection techniques standard for benzo[a]pyrene detection in food). Before HPLC analysis, the *Camellia* seed oil was filtered through a chromatography column loaded with 22 g of alumina and 8.5 g of anhydrous sodium sulfate for purification. About 0.4 mg of the *Camellia* seed oil was weighed into a beaker and dissolved in 2 mL of petroleum ether. The sample solution was then added to the chromatography column and eluted with 80 mL of petroleum ether (flow rate of 1 mL/min). The eluent was dried with nitrogen. The residue was dissolved in 100 μL of acetone, mixed by vortex, and filtered through a 0.45-μm membrane filter before HPLC analysis. Qualitative analysis was performed using the retention time of standard benzo[a]pyrene, and quantitative calculation was performed using a standard curve. The peak areas of the sample in the chromatograms were correlated with the concentrations according to the calibration curve. The linearity was determined by using six standard solutions of benzo[a]pyrene with equidistant concentrations in the range of 0.05–30 μg/kg.

The HPLC chromatographic conditions were as follows: the benzo[a]pyrene was separated with a reversed-phase C-18 column (DiKMA, China) (250 × 4.6 mm, 5 μm). The mobile phase was a mixture of acetonitrile:water (88:12, v/v), and the flow rate was 1.0 mL/min. The benzo[a]pyrene was detected by a fluorescence detector at an excitation wavelength of 384 nm and an emission wavelength of 406 nm.

### Statistical Analysis

One-way analysis of variance was used to show significant differences among the different treatments and the least significant differences were calculated at *p* = 0.05 level. All assays were carried out in triplicate and the results were expressed as mean values ± standard deviations.

## Results and Discussion

### Optimization of Continuous Subcritical Fluid Extraction

Based on the single factor experiments, an orthogonal design L_9_(3^4^) was conducted to optimize the extraction pressure, time, temperature and bulk density (Table 2). Based on the *R* value, the effect of extraction variables on extraction yield decreased in the following order: bulk density (D) >time (B) >pressure (A) >temperature (C). The bulk density had a significant effect on the yield of the *Camellia* seed oil. Based on the *K* values of the variables, the potential highest extraction yield was expected to be obtained at a bulk density of 0.7 kg/L, time of 50 min, pressure of 0.5 MPa and temperature of 45 °C. Under these conditions, three confirmatory tests were carried out. The average extraction yield was 99.12 ± 0.20 %. Therefore, from a long-term perspective, continuous subcritical *n*-butane fluid extraction could be an alternative to the extraction of *Camellia* seed oil.

**Table 2:** Results obtained under the experimental conditions using the L_9_(3^4^) orthogonal design

Trial	Pressure (A, MPa)	Time (B, h)	Temperature (C, °C)	Bulk density (D, kg/L)	Extraction yield (%)
1	1	1	1	1	97.71 ± 0.32
2	1	2	2	2	97.75 ± 0.39
3	1	3	3	3	98.1 ± 0.21
4	2	1	2	3	97.94 ± 0.93
5	2	2	3	1	96.76 ± 0.77
6	2	3	1	2	98.82 ± 0.86
7	3	1	3	2	98.23 ± 1.14
8	3	2	1	3	96.11 ± 0.91
9	3	3	2	1	96.34 ± 0.44
k_1_	96.91	97.75	97.56	96.94	
k_2_	97.84	97.96	97.34	98.27	
k_3_	97.85	96.89	97.7	97.4	
R	0.94	1.07	0.35	1.33	

### Analysis of Fatty Acids

The fatty acid composition of *Camellia* seed oil produced *by* continuous subcritical *n*-butane fluid was analysed by GC–MS. The area normalization method was used to calculate the relative content of the fatty acids (Table 3). The major fatty acids of the *Camellia* seed oil were oleic acid, hexadecanoic acid, and linoleic acid. Oleic acid (73.12 ± 0.40 %) was the principal unsaturated fatty acid, followed by linoleic acid (9.15 ± 0.03 %). Palmitic acid (10.38 ± 0.05 %) was the predominant saturated fatty acid, followed by stearic acid (2.57 ± 0.40 %). The unsaturated fatty acids represented 83.78 ± 0.03 % of the total, including 73.98 ± 0.11 % monounsaturated fatty acids (MUFA) and 9.8 ± 0.04 % polyunsaturated fatty acids (PUFA). Studies have shown that MUFA and PUFA have many important physiological functions, such as reducing coronary heart disease risks [8], stabilizing the integrity of the cell membrane [9], and suppressing arthritis-associated inflammation [10]. The nutrient and healthcare functions of *Camellia* seed oil will become a research hotspot in the future because of superior fatty acid composition.

**Table 3:** Fatty acid composition of *Camellia* seed oil extracted by continuous subcritical *n*-butane fluid extraction

No.	Fatty acids	Retention time	Molecular formula	Chain	Relative content (%)
1	Myristic acid	14.98	C_14_H_28_O_2_	C14:0	0.57 ± 0.06
2	Hexadecenoic acid	16.24	C_16_H_30_O_2_	C16:1	0.12 ± 0.01
3	Palmitic acid	17.9	C_16_H_32_O_2_	C16:0	10.38 ± 0.05
4	Heptadecenoic acid	19.29	C_17_H_32_O_2_	C17:1	0.24 ± 0.02
5	Linoleic acid	21.41	C_18_H_32_O_2_	C18:2	9.15 ± 0.03
6	Oleic acid	21.96	C_18_H_34_O_2_	C18:1	73.12 ± 0.40
7	Octadecanoic acid	22.33	C_18_H_36_O_2_	C18:0	2.57 ± 0.04
8	Linolenic acid	23.09	C_18_H_30_O_2_	C18:3	0.12 ± 0.00
9	Arachidonic acid	23.74	C_20_H_32_O_2_	C20:4	0.28 ± 0.05
10	Eicosenoic acid	25.78	C_20_H_38_O_2_	C20:1	0.5 ± 0.01
11	Eicosanoic acid	26.14	C_20_H_40_O_2_	C20:0	0.33 ± 0.00
12	Others	26.29	C_15_H_22_O_10_	C15:4	0.24 ± 0.07
14		26.41	C_18_H_36_O_4_	C18:0	0.02 ± 0.00
15		27.51	C_27_H_42_O_4_	C27:6	0.01 ± 0.00
	Saturated fatty acids				13.89 ± 0.21
	Unsaturated fatty acids				83.78 ± 0.03
	MUFA				73.98 ± 0.11
	PUFA				9.8 ± 0.04

### Physicochemical Properties

The physicochemical properties of an oil provide information regarding its structural stability and quality [11]. The physicochemical properties of *Camellia* seed oil obtained by continuous subcritical *n*-butane fluid are listed in Table 4. The iodine value (83.20 ± 0.21 gI/100 g), saponification value (154.81 ± 2.00 mg KOH/g), refractive index (1.46 ± 0.06, at 25 °C), freezing-point (−8.00 ± 0.10 °C), unsaponifiable matter (5.00 ± 0.40 g/kg), refrigeration test (transparent, at 0 °C for 5.5 h) and smoke point (215.00 ± 1.00 °C) met the Chinese national standards fora first-class solvent extracted oil based on GB 11765-2003. Although the acid value of 1.24 ± 0.03 mg KOH/g was higher than the value (≤0.25 mg KOH/g) in GB11765-2003, it met the requirement of GB 2716-2005 (4.0 mg KOH/g). Moreover, the higher acid value, probably caused by the long preservation time of *Camellia* seeds, could be reduced to 0.13 ± 0.02 mg KOH/g via molecular distillation. These physicochemical indexes show the possibility of obtaining a high-quality, high-grade, edible *Camellia* seed oil via continuous subcritical *n*-butane fluid extraction.

**Table 4:** Physicochemical properties of *Camellia* seed oil extracted by continuous subcritical *n*-butane fluid extraction

Physicochemical indexes	*Camellia* seed oil extracted by continuous subcritical *n*-butane fluid	National standard of a first-class solvent extraction oil (China)
Iodine value (gI/100 g)	83.20 ± 0.21	≤85
Saponification value (mg KOH/g)	154.81 ± 2.00	≤185
Refractive index (25 °C)	1.46 ± 0.06	1.460–1.464
Freezing point (°C)	−8.00 ± 0.10	−5 to −10
Unsaponifiable matter (g/kg)	5.00 ± 0.40	≤15
Refrigeration test (0 °C, 5.5 h)	Clear, transparent	Clear, transparent
Smoking point (°C)	215.00 ± 1.00	≥215
Acid value (KOH mg/g)	1.24 ± 0.03	≤0.25

### Benzo[a]pyrene Content

**Fig. 1:**
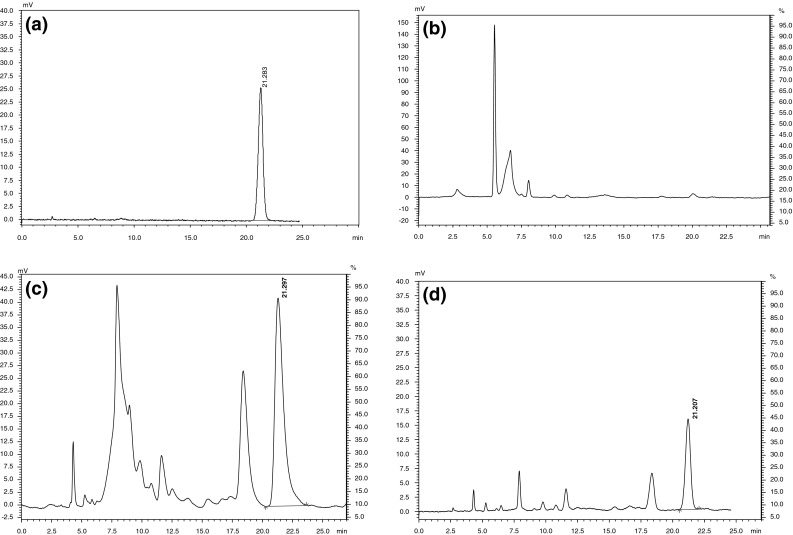
The analysis of benzo[a]pyrene content in different *Camellia* seed oils by HPLC. **a** HPLC chromatogram of a standard benzo[a]pyrene: the retention time was 21.28 min, **b** the analysis of benzo[a]pyrene content in *Camellia* seed oil extracted using continuous subcritical *n*-butane fluid: Benzo[a]pyrene was not found, **c** the analysis of benzo[a]pyrene content in crude *Camellia* seed oil extracted using hot pressing extraction: 26.55 μg/kg, **d** the analysis of benzo[a]pyrene content in refined *Camellia* seed oil extracted using hot pressing extraction 5.69 μg/kg

A number of polycyclic aromatic hydrocarbons (PAH) have been reported to be genotoxic carcinogens. Benzo[a]pyrene, a PAH, has become the focus of attention in *Camellia* seed oil, and researchers have found that different production processes had a great effect on benzo[a]pyrene content in *Camellia* seed oil [6]. The benzo[a]pyrene content in continuous subcritical *n*-butane fluid extraction and hot press extraction of *Camellia* seed oil (crude *Camellia* seed oil and refined *Camellia* seed oil) are shown in Fig. 1. No benzo[a]pyrene was present in *Camellia* seed oil extracted using the continuous subcritical *n*-butane fluid process, while a benzo[a]pyrene content of 26.55 ± 0.70 μg/kg was obtained in crude *Camellia* seed oil and 5.69 ± 0.04 μg/kg was obtained in refined *Camellia* seed oil extracted using a hot press process.

The major cause of excessive benzo[a]pyrene content in *Camellia* seed oil produced by the hot press method is the high temperature employed during extraction, whereas the subsequent refining process, bleaching and winterization, may lead to a reduction in the benzo[a]pyrene content. Because continuous subcritical *n*-butane extraction was carried out at the relatively mild temperature of 45 °C, there was no oil cracking, and therefore no benzo[a]pyrene formation. By using the simple processing method of continuous subcritical *n*-butane extraction, an oil of sufficiently high quality may be obtained that would obviate the need for further refining.

## Conclusions

In this study, *Camellia* seed oil was produced by continuous subcritical *n*-butane fluid extraction. The composition of fatty acids, physicochemical properties, and benzo[a]pyrene content of *Camellia* seed oil were analyzed. Based on the results, the best conditions obtained for continuous subcritical *n*-butane fluid extraction were a bulk density of 0.7 kg/L, a time of 50 min, a pressure of 0.5 MPa, and a temperature of 45 °C. The major fatty acids of *Camellia* seed oil produced by continuous subcritical *n*-butane fluid included oleic acid (73.12 ± 0.40 %), palmitic acid (10.38 ± 0.05 %) and linoleic acid (9.15 ± 0.03 %); unsaturated fatty acids accounted for 83.78 ± 0.03 % of the total fatty acids. Eight main physicochemical properties reflecting the essential characteristics of *Camellia* seed oil produced by continuous subcritical *n*-butane fluid extraction were also analyzed. In particular, benzo[a]pyrene was not found in *Camellia* seed oil produced by continuous subcritical *n*-butane fluid extraction. The benzo[a]pyrene content in crude Camellia seed oil was 26.55 ± 0.70 and 5.69 ± 0.04 μg/kg in refined *Camellia* seed oil extracted using hot press extraction. The experimental results demonstrated that continuous subcritical *n*-butane fluid extraction is a simple process technology, by which a safe and high-quality *Camellia* seed oil could be produced.
